# Targeting the glucagon receptor improves cardiac function and enhances insulin sensitivity following a myocardial infarction

**DOI:** 10.1186/s12933-019-0806-4

**Published:** 2019-01-09

**Authors:** Qutuba G. Karwi, Liyan Zhang, Cory S. Wagg, Wang Wang, Manoj Ghandi, Dung Thai, Hai Yan, John R. Ussher, Gavin Y. Oudit, Gary D. Lopaschuk

**Affiliations:** 1 Cardiovascular Research Centre, 423 Heritage Medical Research Centre, University of Alberta, Edmonton, AB T6G 2S2 Canada; 2 Department of Pharmacology, College of Medicine, University of Diyala, Diyala, Iraq; 3 REMD Biotherapeutics Inc., Camarillo, CA USA; 4 Cosci-REMD Biotherapeutics Inc, Beijing, China

**Keywords:** Glucagon, Insulin signalling, Glucose oxidation, Insulin sensitivity, Branched chain amino acids, Myocardial infarction

## Abstract

**Background:**

In heart failure the myocardium becomes insulin resistant which negatively influences cardiac energy metabolism and function, while increasing cardiac insulin signalling improves cardiac function and prevents adverse remodelling in the failing heart. Glucagon’s action on cardiac glucose and lipid homeostasis counteract that of insulin’s action. We hypothesised that pharmacological antagonism of myocardial glucagon action, using a human monoclonal antibody (mAb A) against glucagon receptor (GCGR), a G-protein coupled receptor, will enhance insulin sensitivity and improve cardiac energy metabolism and function post myocardial infarction (MI).

**Methods:**

Male C57BL/6 mice were subjected to a permanent left anterior descending coronary artery ligation to induce MI, following which they received either saline or mAb A (4 mg kg^−1^ week^−1^ starting at 1 week post-MI) for 3 weeks.

**Results:**

Echocardiographic assessment at 4 weeks post-MI showed that mAb A treatment improved % ejection fraction (40.0 ± 2.3% vs 30.7 ± 1.7% in vehicle-treated MI heart, p < 0.05) and limited adverse remodelling (LV mass: 129 ± 7 vs 176 ± 14 mg in vehicle-treated MI hearts, p < 0.05) post MI. In isolated working hearts an increase in insulin-stimulated glucose oxidation was evident in the mAb A-treated MI hearts (1661 ± 192 vs 924 ± 165 nmol g dry wt^−1^ min^−1^ in vehicle-treated MI hearts, p < 0.05), concomitant with a decrease in ketone oxidation and fatty acid oxidation rates. The increase in insulin stimulated glucose oxidation was accompanied by activation of the IRS-1/Akt/AS160/GSK-3β pathway, an increase in GLUT4 expression and a reduction in pyruvate dehydrogenase phosphorylation. This enhancement in insulin sensitivity occurred in parallel with a reduction in cardiac branched chain amino acids content (374 ± 27 vs 183 ± 41 µmol g protein^−1^ in vehicle-treated MI hearts, p < 0.05) and inhibition of the mTOR/P70S6K hypertrophic signalling pathway. The MI-induced increase in the phosphorylation of transforming growth factor β-activated kinase 1 (p-TAK1) and p38 MAPK was also reduced by mAb A treatment.

**Conclusions:**

mAb A-mediated cardioprotection post-myocardial infarction is associated with improved insulin sensitivity and a selective enhancement of glucose oxidation via, at least in part, enhancing branched chain amino acids catabolism. Antagonizing glucagon action represents a novel and effective pharmacological intervention to alleviate cardiac dysfunction and adverse remodelling post-myocardial infarction.

## Introduction

Following a myocardial infarction (MI) adverse remodelling can occur in the non-infarcted myocardium [1]. Significant perturbations in cardiac energy metabolism can occur during this remodelling process, which can contribute to the severity of heart failure (HF) development [2, 3]. This includes the development of cardiac insulin resistance, similar to what occurs in diabetes [3, 4]. In fact, cardiac insulin resistance is further aggravated in HF associated with diabetes [5, 6]. While it is not clear how this cardiac insulin resistance occurs in HF, a major hallmark of cardiac insulin insensitivity is a marked reduction in mitochondrial insulin-stimulated glucose oxidation [3, 4]. This is of significance, since enhancing insulin sensitivity can effectively reduce the cardiac energy deficit and improve cardiac function in the failing heart [2, 7].

Fatty acid oxidation is a major source of ATP production in the post-MI and failing heart [8–10]. Interestingly, inhibition of fatty acid oxidation can improve cardiac efficiency and contractile function in the post-MI and failing heart [7, 11, 12]. Elevated cardiac ketone oxidation rates are another metabolic signature in the failing heart [13, 14]. This alteration has been suggested to be an adaptive mechanism where ketones might be an extra source of fuel for the failing heart [13, 15]. However, it is not yet known what happens to ketone oxidation rates in post-MI hearts.

Another metabolic derangement in the failing heart is a decrease in the metabolism of branched chain amino acids (BCAA), namely leucine, isoleucine and valine, which lead to their accumulation in the myocardium [16–18]. Impaired BCAA catabolism strongly correlates with the occurrence of insulin resistance not only in the heart [16–19] but also the whole body [20–22]. Emerging evidence suggests that impaired BCAA catabolism aggravates insulin insensitivity post-MI, at least in part, by inhibiting pyruvate dehydrogenase (PDH), the rate-limiting enzymes of glucose oxidation, and suppressing glucose oxidation in the heart [23]. Defective BCAA metabolism also further aggravates cardiac dysfunction and adverse remodelling following myocardial ischemia/reperfusion injury through triggering the mammalian target of rapamycin (mTOR) signalling pathway [23, 24]. Krüppel-like factor 15 (KLF15) is a direct transcriptional activator of mitochondrial branched chain aminotransferase (BCATm), the first enzymatic step in BCAA catabolism, and can modulate the hypertrophic signalling of mTOR in skeletal muscle [25, 26]. KLF15 is down-regulated by upstream activation of p38 mitogen-activated protein kinase (p38 MAPK), and is also down-regulated in murine models of HF [18] and patients with cardiomyopathy [27, 28], which is in line with the dysregulation of BCAA catabolism. Taken together, improving BCAA catabolism could be a potential therapeutic approach to enhance cardiac insulin sensitivity and limit pathological remodelling post-MI.

Glucagon has important effects on glucose and lipid metabolism, which generally oppose the actions of insulin, and its actions dominate in the setting of insulin resistance [29, 30]. In support of this concept, antagonising glucagon receptor (GCGR) signalling can improve insulin sensitivity in experimental models of diabetes and obesity [31]. It has also been recently reported that exogenous glucagon administration is associated with increased mortality in a murine model of MI in a p38 MAPK-dependent manner [32]. Interestingly, cardiac-specific GCGR deletion (*Gcgr*^CM−/−^) enhances survival and reduces adverse left ventricular (LV) remodelling post-MI [32]. This cardioprotection was proposed to be due to an inhibition of fatty acid oxidation in the *Gcgr*^CM−/−^ mouse hearts. Whether this cardioprotection was associated with improvements in insulin-stimulated glucose oxidation is not clear. Furthermore, the impact of glucagon signalling on BCAA catabolism has not yet been investigated.

As antagonising glucagon signalling is shown to improve insulin sensitivity of the whole body, we investigated the potential cardioprotection of mAb A, a human IgG2 monoclonal antibody against the GCGR receptor, and whether it can enhance cardiac insulin sensitivity and, as a result, improve cardiac function post-MI. In this study, we provide a novel and clinically translatable approach using mAb A to improve cardiac function and reduce adverse remodelling post-MI in a mouse model of MI. mAb A-mediated cardioprotection involved enhancing insulin signalling and glucose oxidation in the post-MI heart.

## Methods

### Animals

All procedures and studies were conducted in accordance with University of Alberta Health Sciences Animal Welfare Committee and the Canadian Council of Animal Care (AUP-00000288). Male C57BL/6 mice (8–10 weeks old) were purchased from Charles River Laboratories, Wilmington, MA. Animals had free access to regular chow and drinking water ad libitum.

### Murine model of myocardial infarction

Myocardial infarction was induced in vivo by permanent ligation of the proximal left anterior descending (LAD) coronary artery, as previously reported [8, 33]. Briefly, animals were anaesthetised with an intraperitoneal injection of ketamine/xylazine (100 mg kg^−1^/10 mg kg^−1^), then intubated using a 14-gauge polyethylene catheter and ventilated with room air using a mouse ventilator. When the anaesthesia reached a surgical plane, the animals underwent a left thoracotomy in the fifth intercostal space to expose the heart. The pericardium was dissected, and a 7-0 silk suture was placed around the LAD and permanently tightened. Sham animals had the same surgical procedure and the suture was placed underneath the coronary artery but was not ligated. The muscle and skin were closed using 6-0 silk suture, following which the animal received a subcutaneous injection of analgesia (Metacom 1 mg kg^−1^, Boehringer Ingelheim Vetmedica Inc., Germany) and allowed to fully recover in a recovery cage. We commence the treatment protocol 1 week after the surgery to allow the myocardial infarction to evolve.

### Treatment protocol

**Fig. 1:**
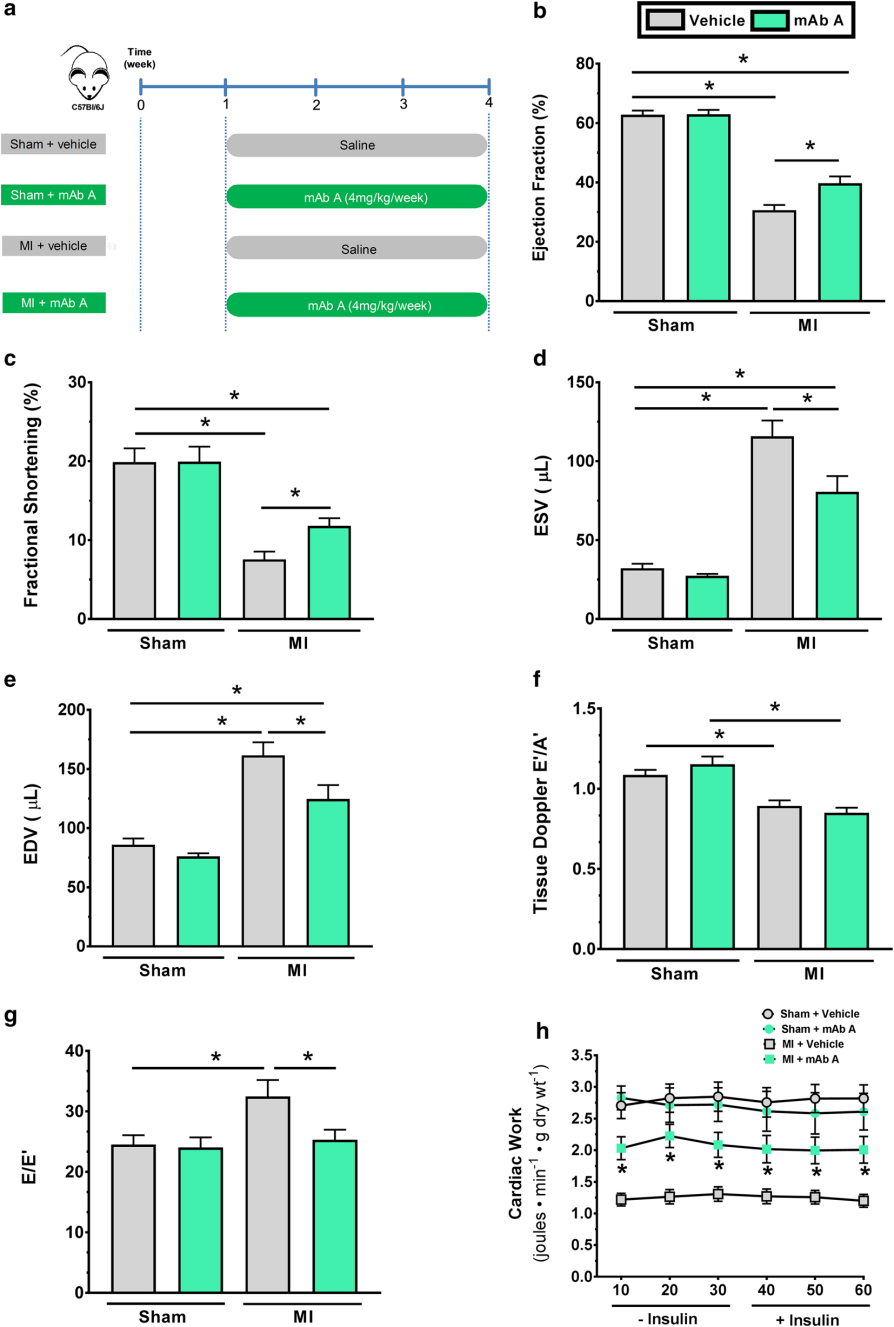
mAb A treatment improves ejection fraction and fractional shortening post myocardial infarction (MI). **a** Schematic of the study design and treatment protocol. Mice were randomised to undergo either sham or left anterior descending (LAD) coronary artery ligation surgery. A week after the surgery, mice in both groups were randomly assigned to receive either saline (vehicle) or mAb A treatment (4 mg kg^−1^ week^−1^, s.c.) for 3 weeks. Echocardiographic analysis for sham and MI mice including: **b** ejection fraction (%), **c** fractional shortening (%), **d** end systolic volume (ESV) (µL), **e** end diastolic volume (EDV) (µL), **f** tissue doppler E′/A′ and **g** E/E, **h** ex vivo cardiac work (joules min^−1^ g dry wt^−1^). Data are reported as mean ± SEM. *p < 0.05. n = 12–18

A week after the surgery, animals in the sham and MI groups were randomised to receive a subcutaneous injection of either vehicle (saline) or mAb A (4 mg kg^−1^ day^−1^) for 3 weeks (Fig. 1a). mAb A was provided by REMD Biotherapeutics Inc. (Camarillo, California, USA). The activity of mAb A against mouse GCGR has previously been characterised [34].

### Assessment of the cardiac function in vivo by echocardiography

Cardiac function was evaluated at the end of the 4 weeks post-MI period using echocardiography (Vevo 770, Visualsonic, Toronto). Left ventricle (LV) dimensions were measured using a modified version of the leading-edge method of the American Society for Echocardiography using three consecutive cycle of M-mode tracing. Left ventricular (LV) end systolic and diastolic volume (LVESV and LVEDV) were also estimated with left ventricular internal diameter tracing by positioning the M-mode cursor perpendicular to the left ventricular anterior and posterior wall. Systolic and diastolic peak velocities were measured at the mitral valve level, and LV filling velocity was assessed using the ratio of early (E) and late (A) mitral inflow velocities as well as the ratio between early mitral inflow velocity and mitral annular early diastolic velocity (E/E′). Ejection fraction (%EF) was also calculated using Simpson’s measurements. The analysis was carried out in a blind fashion. A random sample of pre-analysed clips/samples were re-analysed routinely by the same experimentalist to minimize “intra-observer variability”. In addition, a random sample of pre-analyzed clips/samples were also re-analysed routinely by another experimentalist to minimize the “inter-observer variability”.

### Blood glucose, free fatty acid, ketone and branched chain amino acids measurements

Blood samples from the tail vein were collected from mice in the fed state at the end of the 4 weeks study period and the plasma glucose levels were measured using a glucometer. Circulating levels of free fatty acids, ketones and BCAA were also measured using ELISA kits from Sigma-Aldrich (Cat: 11383175001), Wako diagnostics (Cat: 415-73301 and 411-73301) and BioVision (Cat: K564100), respectively, following the manufacturer instructions.

### Isolated working heart perfusions

Fed mice were euthanised with an intraperitoneal injection of pentobarbital (60 mg kg^−1^), hearts were collected and aerobically perfused with Krebs–Henseleit buffer (KHB), contains (in mmol L^−1^) 2.5 Ca^2+^, 5 glucose, 0.6 β-hydroxybutyrate (β-OHB), and 0.8 palmitate pre-bound to 3% fatty acid free bovine serum albumin as previously reported [2, 35, 36]. Briefly, the heart was perfused in a working heart setting, where the system was sealed to allow a quantitative measurement of ^14^CO_2_ generated from ^14^C-labelled carbon substrates. The system also allowed the measurement of ^3^H_2_O generated from ^3^H-labelled energy substrates. One set of hearts from each experimental group was perfused with [5-^3^H] glucose and [U-^14^C] glucose to simultaneously measure glycolysis and glucose oxidation rates, respectively, while another set of hearts was perfused with [9,10-^3^H] palmitate and [3-^14^C] β-OHB to measure palmitate and β-OHB oxidation rates simultaneously. The hearts were perfused for 30 min, after which 100 µU/mL insulin was added to the heart perfusate and the heart was perfused for an additional 30 min. Cardiac function was monitored throughout the ex vivo experiments and cardiac work was calculated from left ventricular developed pressure and cardiac output. At the end of perfusion protocol, the heart was snap frozen with tongs cooled to the temperature of liquid nitrogen and stored at − 80 °C for further molecular characterisation.

### Immunoblotting analysis

Protein samples (30 µg/well) were loaded onto 10% SDS-PAGE to semi-quantify the changes in the regulatory enzymes and kinases of the cardiac metabolism as described previously [37, 38]. The membrane was then incubated with different primary antibodies (1:1000), except for molecular weight > 100 kDa where (1:500) was used, and correspondent secondary antibodies (1:5000), based on the protein of interest. The following antibodies were used: with the following primary antibodies: mTOR (Cell Signaling, catalog 2983), phospho-mTOR (Cell Signaling, catalog 2983S), P70S6K (Cell Signaling, catalog 9205), phospho-P70S6K (Cell Signaling, catalog 9205S), SERCA2A (Cell Signaling, catalog 4388S), phospho-IRS-1 (Milipore, catalog 09-433), IRS-1 (Cell Signaling, catalog 2382S), phospho-Akt (Cell Signaling, catalog 9271S), Akt (Cell Signaling, catalog 9272S), phospho-GSK-3β (Cell Signaling, catalog 9322S), GSK-3β (Cell Signaling, catalog 9321S), phospho-AS160 (Cell Signaling, catalog 4288S), AS160 (Cell Signaling, catalog 2447S), GLUT4 (Cell Signaling, catalog 2213S), phospho-PDH (Milipore, catalog ABS204), PDH (Cell Signaling, catalog 3205S), BCATm (Cell Signaling, Catalog 9432S), KLF15 (Santa Cruz, catalog sc-271675), phospho-p38 (Cell Signaling, catalog 9211S), p38 (Cell Signaling, catalog 9212S), phospho-TAK1 (Cell Signaling, catalog 4508S), TAK1 (Cell Signaling, catalog 4505S), phospho-AMPK (Cell Signaling, catalog 2535S), AMPK (Cell Signaling, catalog 2603S), phospho-ACC (Milipore, catalog 07-303), ACC (Cell Signaling, catalog 3662S), MCD (Abcam, catalog ab95945), acetyl lysin (Cell Signaling, catalog 9441S), β-HAD (Abcam, catalog ab37673), LCAD (Abcam, catalog ab129711), BDH1 (Abcam, catalog ab68321), SCOT (Abcam, catalog ab70413). Membranes were then incubated with the appropriate secondary antibodies (goat anti-rabbit, catalog 7074P2; goat anti-mouse, catalog 31430; goat anti-chicken, catalog A16054) for 1–2 h. The protein bands were visualised using the Amersham enhanced chemiluminescence (Cell Signalling Technologies, Danvers, Massachusetts, USA). Densitometry was conducted in a blind fashion using ImageJ program (1.48v, National Institutes of Health USA) and all protein bands intensity were normalised to α-tubulin which served as an internal control (loading control).

### Immunoprecipitation

All procedures were conducted at 4 °C to preserve the protein integrity. Tissue lysate (300 µg) was incubated with 50 µL of A/G PLUS-Agarose beads (Santa Cruz Biotechnology) for 4 h for pre-cleaning, then centrifugated at 16,000*g* for 5 min and the supernatant (cleaned lysate) was collected. The protein sample was then incubated with anti-acetyl-lysine antibody (2 µL, Millipore Sigma) overnight then 30 µL of A/G PLUS-Agarose beads was added to the solution and incubated for 6 h. Samples were centrifugated at 5000*g* for 5 min and the pellet was washed three times with the homogenisation buffer. Laemmli buffer was then added to the pellet and the mixture, heated at 95 °C for 5 min then centrifuged at 15,000*g* for 15 min. The supernatant was carefully eluted, and the proteins were blotted following the same protocol for immunoblotting in the previous section. Negative control sample (−C) contained A/G PLUS-Agarose beads and anti-acetyl-lysine antibody with no protein sample, while positive control sample (+C) contained protein sample and Laemmli buffer which has not been incubated with either A/G PLUS-Agarose beads or anti-acetyl-lysine antibody.

### Statistical analysis

Data are reported as mean ± SEM and were subjected to Kolmogorov–Smirnov test for normal distribution. An unpaired, 2-tailed Student *t* test was used to compare between two experimental groups, while one-way or two-way ANOVA were used for multiple comparisons with Bonferroni as a post hoc test. The difference between groups was considered significant when the p-value < 0.05. GraphPad Prims^®^ 7 software was used to draw all the figure and to conduct the statistical analysis.

## Results

### mAb A treatment improves cardiac function in the post-MI heart

Mice were studied 4 weeks after being subjected to a permanent LAD ligation (Fig. 1a). A significant reduction in the % ejection fraction (%EF) and fractional shortening (FS%) was observed by 4 weeks post-MI in vehicle-treated mice (30.7 ± 1.8% vs 62.8 ± 1.4% and 7.6 ± 1.0% vs 20.0 ± 1.8% in the sham mice, p < 0.05, respectively, Fig. 1b, c). However, a 3-week treatment with mAb A significantly improved the %EF post-MI (39.71 ± 2.3%, p < 0.05, Fig. 1b) and fractional shortening (11.8 ± 1.0%, p < 0.05 Fig. 1c). In addition, myocardial dilation was observed in the post-MI mice hearts, with mAb A treatment attenuating this dilation (Fig. 1 d, e). No detectable changes in tissue doppler of the mitral valve E′/A′ ratio were in seen in mAb A treated mice compared to vehicle-treated mice (Fig. 1f), although E/E′ ratio was significantly improved in the mAb A-treated MI mice (28.4 ± 2.8 mm s^−1^ vs 32.5 ± 2.7 mm s^−1^ vehicle-treated post MI mice, p < 0.05, Fig. 1g). Consistent with the in vivo heart function, ex vivo cardiac work in the isolated working hearts from post-MI mice was decreased relative to sham mice (Fig. 1h) and was also improved by mAb A treatment (Fig. 1h).

### mAb A treatment inhibits cardiac hypertrophy and the mTOR signalling pathway

**Fig. 2:**
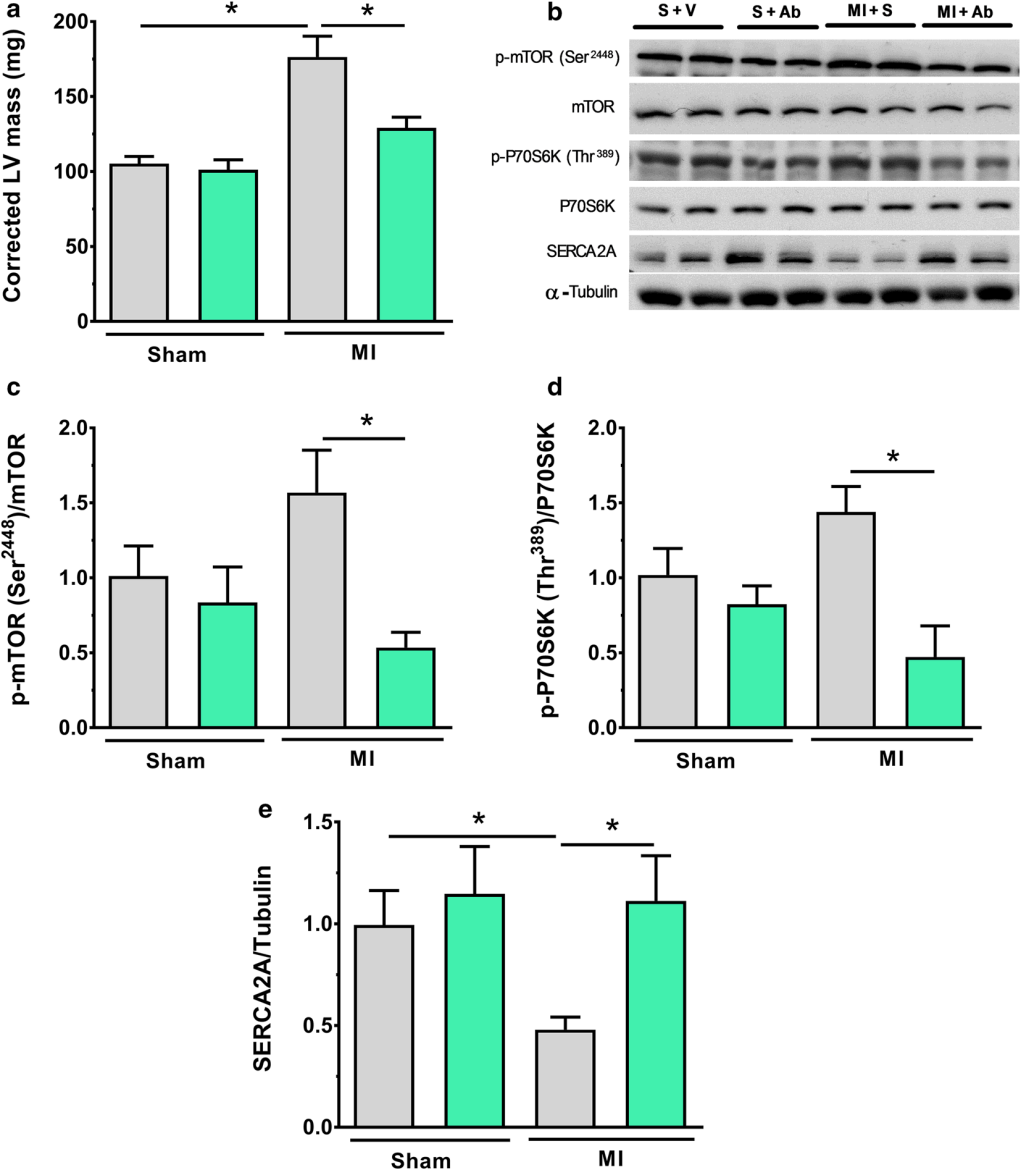
Abrogated cardiac remodelling by mAb A treatment is associated with inhibition of mTOR signalling pathway post-MI. **a** In vivo corrected left ventricle (LV) mass (mg). **b** Representative blots for mTOR signalling pathway. Densiometric analysis for **c** phosphorylated mammalian target of rapamycin (p-mTRO^Ser2448^)/mTOR, **d** phosphorylated ribosomal protein S6 kinase (p-P70S6K^Thr389^)/P70S6K, **e** sarco/endoplasmic reticulum Ca^2+^-ATPase (SERCA2A)/α-tubulin. Protein bands were normalised for either to their correspondent total protein band or to α-tubulin bands for intra-experiment variation. Data are represented as mean ± SEM. *p < 0.05. n = 6/group. *S* sham, *V* vehicle, *Ab* mAb A, *MI* myocardial infarction

Cardiac hypertrophy was observed in vehicle-treated MI mice compared to vehicle-treated sham mice (Fig. 2a). This hypertrophy was accompanied by the activation of cardiac mTOR (Fig. 2c), and an increased phosphorylation of P70S6K^Thr389^, a downstream effector of mTOR (Fig. 2d). mAb A treatment limited cardiac hypertrophy, an effect which was associated with abrogating mTOR/P70S6K signalling (Fig. 2b–d). In addition, a decrease in cardiac SERCA2A expression was observed in the post-MI mice (Fig. 2e), which has previously been observed in hypertrophied hearts [39, 40]. This decreased expression of SERCA2A post-MI was prevented with mAb A treatment (Fig. 2e), consistent with the decrease in hypertrophy observed in these hearts (Fig. 2a).

### mAb A treatment mitigates the hyperglycaemia and hyperketonemia in the post-MI mice

**Fig. 3:**
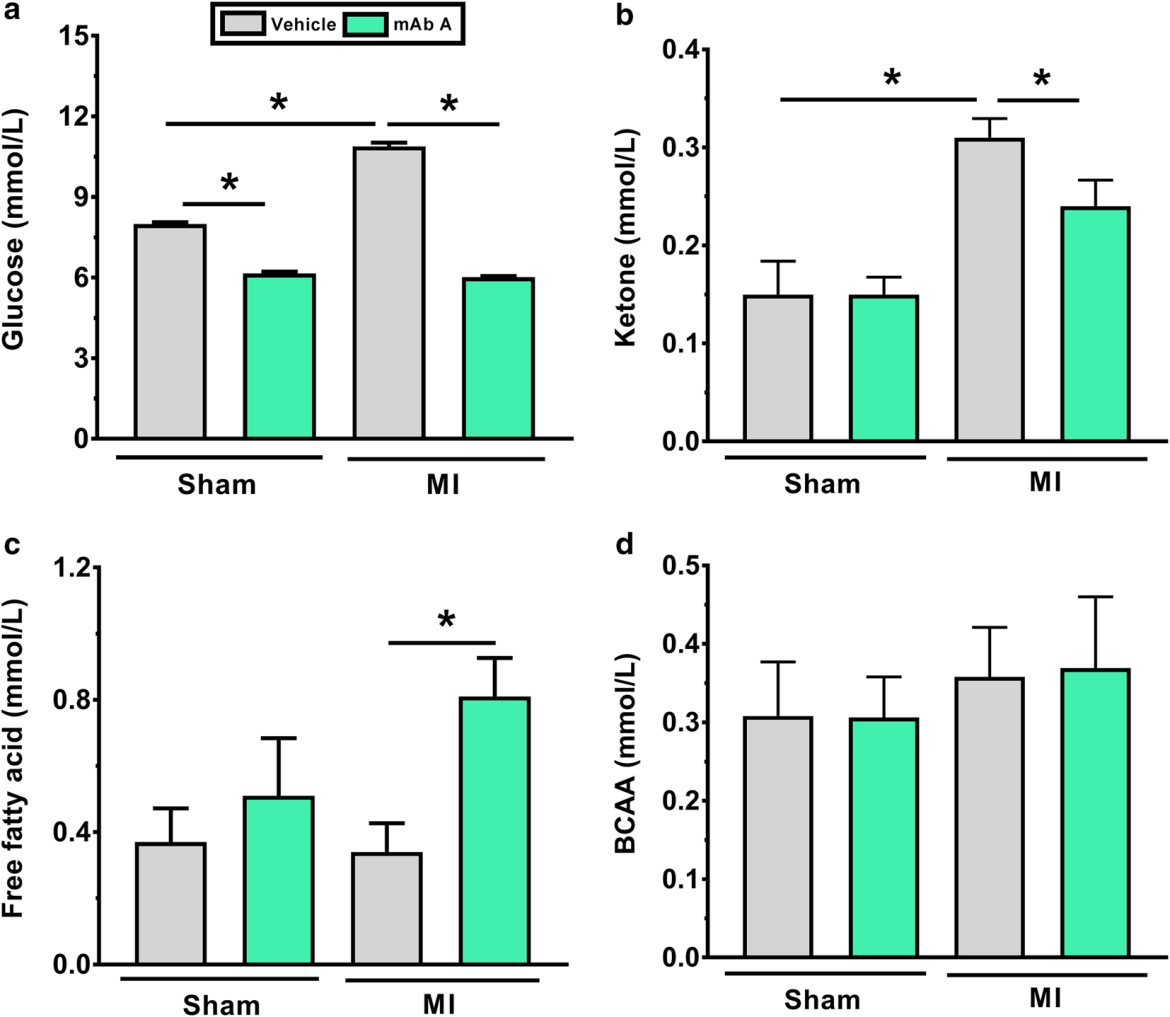
mAb A treatment improves glycemic control post-MI. Blood samples were collected from the tail vein at the end of the treatment protocol to measure **a** blood glucose level, **b** ketone bodies level, **c** free fatty acid level and **d** branched chain amino acids (BCAA). Data are reported as mean ± SEM. *p < 0.05. n = 7–10

Vehicle-treated post-MI mice had a significant increase in blood glucose levels (Fig. 3a) and ketone bodies levels (Fig. 3b) compared to sham mice, with no change in circulating fatty acid levels (Fig. 3c) or BCAA levels (Fig. 3d). mAb A treatment was associated with a reduction in glucose level in the sham mice (Fig. 3a). In addition, mAb A treatment markedly reduced glucose and ketone levels in the post-MI mice (Fig. 3a, b). Interestingly, chronic treatment with mAb A did not cause a significant change in circulating free fatty acids in the sham group, although post-MI mice treated with mAb A showed a significant increase in circulating free fatty acids compared to vehicle-treated MI mice (Fig. 3c). mAb A treatment post-MI did not have a significant effect on circulating BCAA compared to vehicle-treated mice (Fig. 3d).

### mAb A treatment enhances glucose oxidation while reducing fatty acid and ketone oxidation in the post-MI heart, results in improving TCA cycle activity

**Fig. 4:**
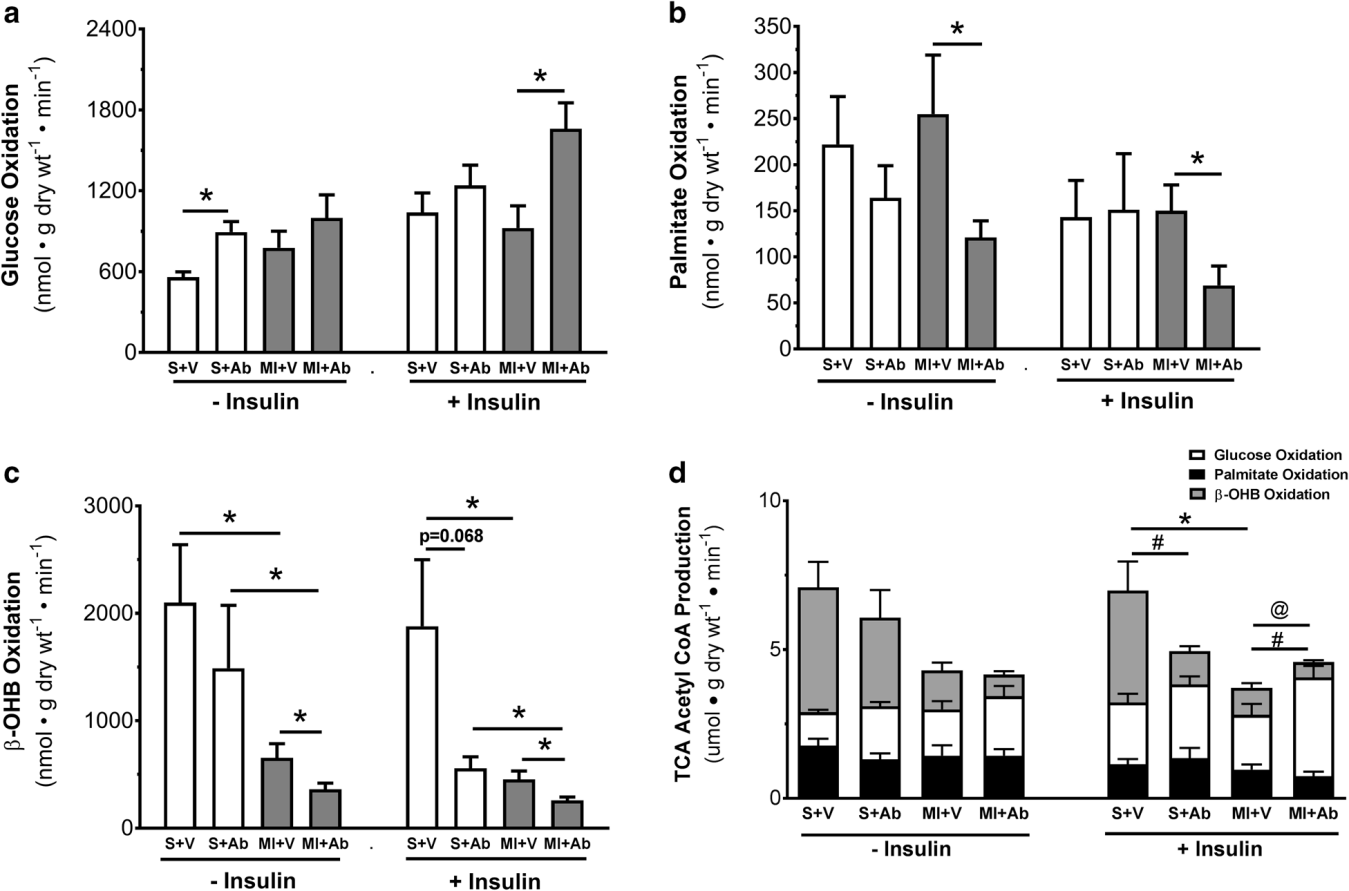
mAb A treatment enhances insulin sensitivity, increases glucose oxidation and inhibits fatty acid oxidation post-MI. In ex vivo isolated working hears, the metabolic profile for sham and MI hearts where characterised including measuring **a** glucose oxidation rates, **b** palmitate oxidation rates, **c** β-hydroxybutyrate (β-OHB) oxidation rates and **d** contribution of each energy substrate to tricarboxylic acid (TCA) cycle acetyl CoA supply, in the presence and absence of insulin. Data are reported as mean ± SEM. *p < 0.05. # indicates significant difference in β-hydroxybutyrate oxidation rates, while @ indicates significant difference in glucose oxidation rates. n = 9–13. *S* sham, *V* vehicle, *Ab* mAb A, *MI* myocardial infarction

To understand whether mAb A-mediated cardioprotection is associated modification of cardiac energy metabolism, we measured glucose, palmitate and β-OHB oxidation rates in isolated working hearts. A marked increase in insulin-stimulated glucose oxidation was observed in the MI hearts post mAb A treatment (1661 ± 192 vs 924 ± 165 nmol g dry wt^−1^ min^−1^ relative to vehicle, p < 0.05, Fig. 4a). Palmitate oxidation rates were not significantly affected by mAb A in the sham group (Fig. 4b), whereas a marked reduction in palmitate oxidation rate was observed in MI hearts post mAb A treatment compared to vehicle in the presence and absence of insulin (Fig. 4b). In addition, β-OHB oxidation rates was suppressed in vehicle-treated MI hearts relative to sham. A further reduction in β-OHB oxidation was also observed in the MI hearts post mAb A treatment, both in the absence or presence of insulin.

There was a significant decrease in mitochondrial acetyl CoA production for the TCA cycle in MI hearts relative to sham hearts, which was significantly enhanced by mAb A treatment (Fig. 4d). Of which, this enhancement was mainly due to a significant increase in the contribution of glucose oxidation as a source of TCA cycle acetyl CoA, in parallel with a reduction in β-OHB oxidation contribution to the TCA cycle acetyl CoA (Fig. 4d).

### mAb A treatment enhances the insulin signalling pathway in the post-MI heart

**Fig. 5:**
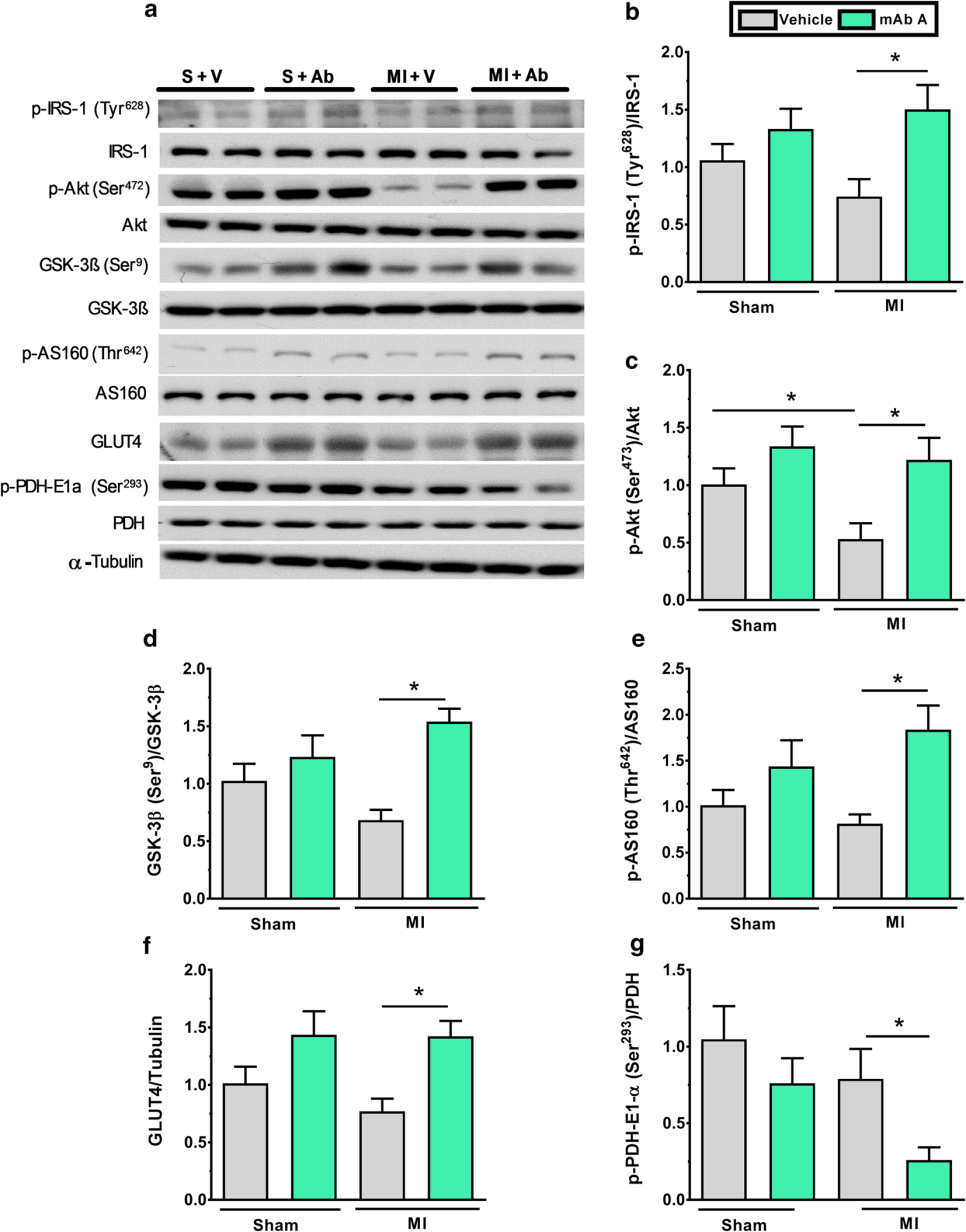
mAb A treatment enhances insulin signalling in the heart post-MI. **a** Representative blots for insulin signalling kinases. Densiometric analysis for **b** phosphorylated insulin receptor substaret-1 (p-IRS-1^Tyr628^)/IRS-1, **c** phosphorylated protein kinase B (p-Akt^Ser473^)/Akt, **d** phosphorylated glycogen synthase kinase-beta (p-GSK-3β^Ser9^)/GSK-3β, **e** phosphorylated Akt substrate 160 (AS160^Thr642^)/AS160 **f** glucose transporter type-4 (GLUT4)/α-tubulin, and **g** phosphorylated pyruvate dehydrogenase (p-PDH-E1α^Ser293^)/PDH. Protein bands were normalised for either to their correspondent total protein band or to α-tubulin bands for intra-experiment variation. *p < 0.05. n = 6/group. *S* sham, *V* vehicle, *Ab* mAb A, *MI* myocardial infarction

To understand if improved cardiac function occurred in conjunction with an altered cardiac insulin signalling, we assessed the IRS/Akt/GSK-3β pathway. mAb A treatment activated IRS-1 at its tyrosine^628^ residue (Fig. 5b). This activation was accompanied by triggering Akt activity and inhibiting GSK-3β activity by increasing its phosphorylation at serine^9^ (Fig. 4c, d), along with an increase in the phosphorylation of AS160^Thr642^ and the expression of GLUT4, the insulin-sensitive glucose transporter (Fig. 5e, f). Furthermore, phosphorylation of pyruvate dehydrogenase (PDH) at its serine^293^ residue, the key enzyme in mitochondrial glucose oxidation, was decreased in the MI hearts post mAb A treatment (which would be expected to increase PDH activity) (Fig. 5g), which supports the observed effect of mAb A increasing cardiac glucose oxidation rates (Fig. 4a).

### mAb A treatment enhances cardiac BCAA catabolism post-MI in parallel with inhibiting the TAK1/p38 MAPK signalling pathway

**Fig. 6:**
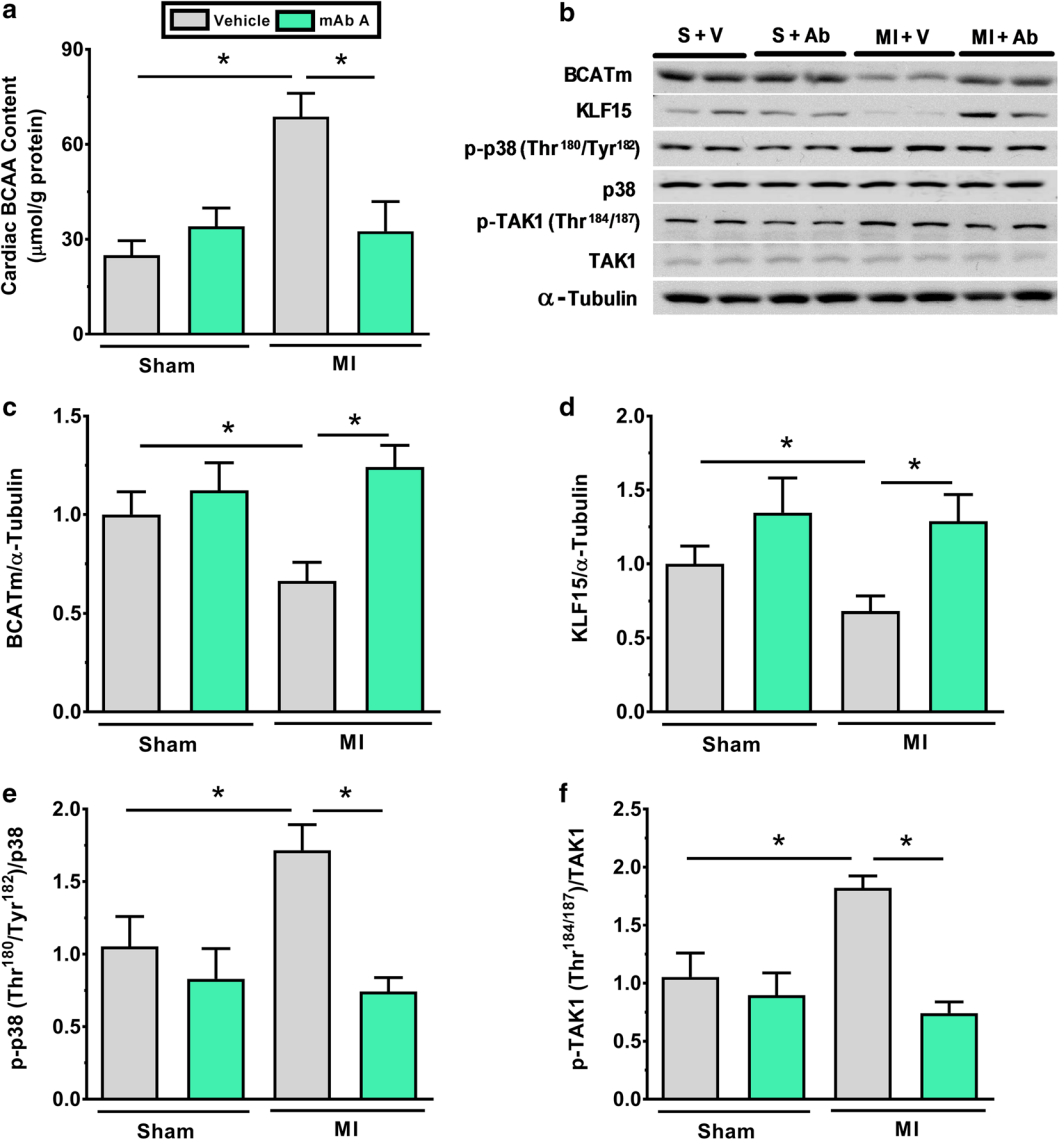
mAb A treatment prevents the accumulation of branched chain amino acids (BCAA) post-MI via enhancing their catabolism. **a** Myocardial BCAA concentration, **b** representative blots for BCAA catabolism enzymes, **c** mitochondrial branched chain aminotransferase (BCATm)/α-tubulin, **d** Krüppel-like factor 15 (KLF15)/α-tubulin, **e** phosphorylated p38 mitogen-activated protein kinases (p-p38 MAPKThr^180^/Tyr^182^)/p38 MAPK, **f** phosphorylated-transforming growth factor beta-activated kinase 1 (p-TAK1 Thr^184/187^)/TAK1. Densitometry were carried out when protein bands were normalised to α-tubulin bands for intra-experiment variation. Data are reported as mean ± SEM n = 6/group. *S* sham, *V* vehicle, *Ab* mAb A, *MI* myocardial infarction

Impaired BCAA catabolism correlates with the occurrence of insulin resistance and muscle hypertrophy through activating the mTOR pathway [21, 41]. Since an improved cardiac insulin signalling was observed in MI hearts post mAb A treatment (Fig. 5) concomitantly with an inhibition in the hypertrophic signal (Fig. 2), we assessed whether these modifications were associated with any alterations in cardiac BCAA catabolism. A marked increase in cardiac BCAA levels were observed in the MI hearts compared to the sham hearts (69 ± 7 vs 25 ± 5 µmol g^−1^ protein, Fig. 6a). A dramatic decrease in cardiac BCAA levels was evident in the MI heart post mAb A treated hearts (33 ± 9 µmol g^−1^, Fig. 6a), suggesting that mAb A treatment may improve cardiac BCAA catabolism. Indeed, the expressions of mitochondrial branched chain aminotransferase (BCATm), a key enzyme in BCAA catabolism, and Krüppel-like factor 15 (KLF15), a major transcriptional regulator of BCAA metabolism, were reduced in the MI hearts relative to sham hearts, while mAb A treatment reversed these changes (Fig. 6c, d). The activity of KLF15 in the heart is regulated via p38 MAPK activity where triggering p38 MAPK activity, in response to transforming growth factor beta-activated kinase 1 (TAK1), can inhibit KLF15 activity [42]. This is significant since the detrimental effects of glucagon in the ischemic heart is shown to be partly mediated through a p38-dependant mechanism [32]. In our study, we found that TAK1/p38 MAPK signal was upregulated in the MI hearts, while mAb A treatment abolished this signal. This suggests an effect of mAb A on enhancing BCAA catabolism through a mechanism which involves inhibition of TAK1/p38 MAPK/KLF15 signalling.

### mAb A treatment reduces fatty acid oxidation by inhibiting 5′AMP-activated protein kinase (AMPK) and malonyl CoA decarboxylase (MCD) in the post-MI heart

**Fig. 7:**
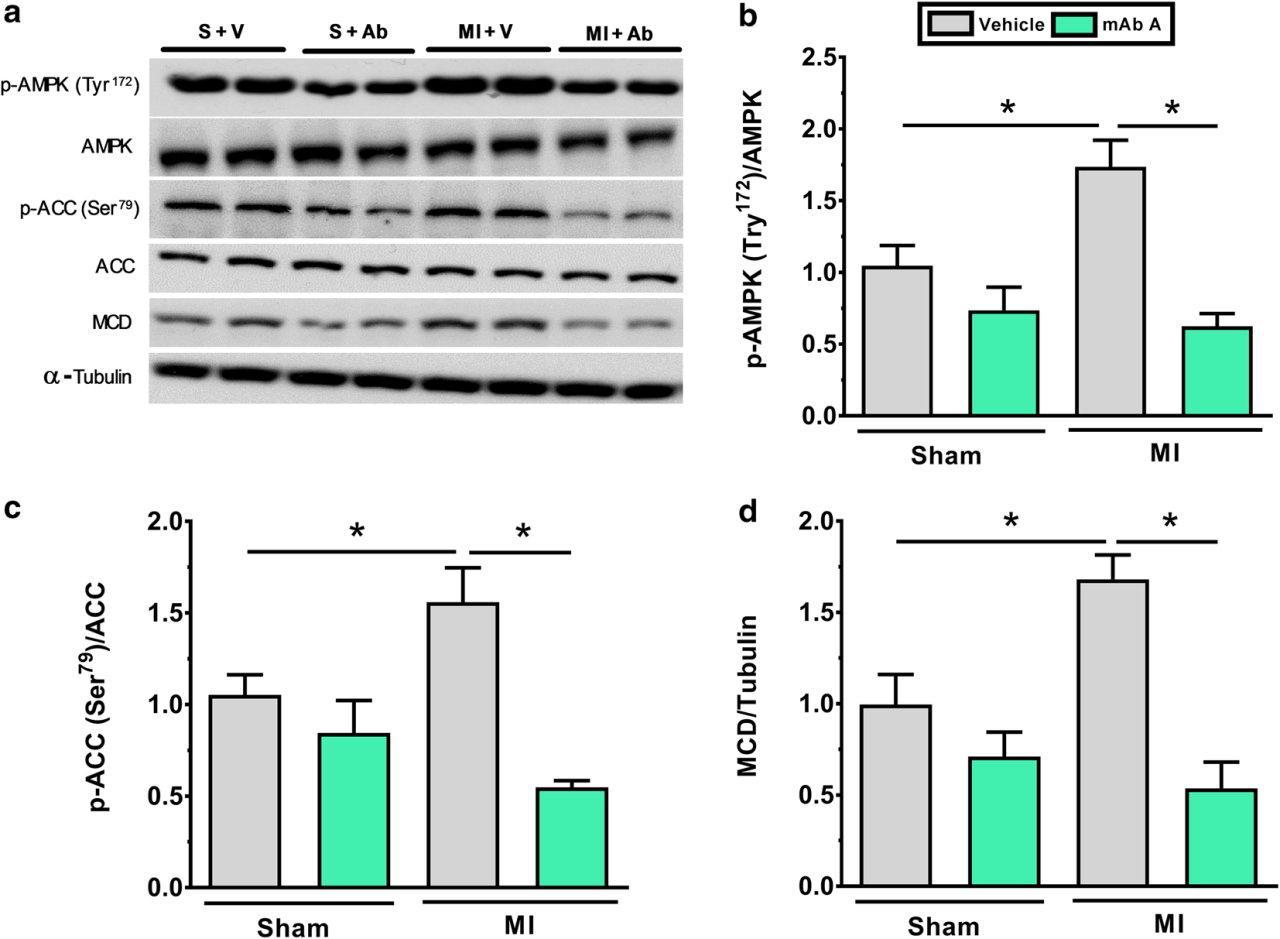
mAb A treatment inhibits AMP-activated protein kinase (AMPK) and acetyl CoA carboxylase (ACC) phosphorylation and increases malonyl CoA decarboxylase MCD expression post-MI. **a** Representative blots for fatty acid regulatory signalling. **a** Densiometric analysis for **b** phosphorylated 5′ AMP-activated protein kinase (p-AMPK^Tyr172^)/AMPK, **c** phosphorylated acetyl CoA carboxylase (p-ACC^Ser79^)/ACC, **d** malonyl CoA decarboxylase (MCD)/α-tubulin. Protein bands were normalised for either to their correspondent total protein band or to α-tubulin bands for intra-experiment variation. *p < 0.05. n = 6/group. *S* sham, *V* vehicle, *Ab* mAb A, *MI* myocardial infarction

5′AMP-activated protein kinase is a key regulator of cardiac fatty acids oxidation in the normal and ischemic heart [43]. This is through, at least in part, by phosphorylating and decreasing the activity of acetyl CoA carboxylase (ACC), which decreases the synthesis of malonyl CoA, thereby stimulating fatty acid oxidation (since malonyl CoA is a potent inhibitor of fatty acid oxidation) [44–46]. We, therefore, assessed if the decrease in fatty acid oxidation rates induced by mAb A treatment in MI hearts (Fig. 4b) is accompanied by alterations in AMPK signalling. In MI hearts a marked increase in the phosphorylation of AMPK at its tyrosine^172^ residue was observed (which increases AMPK activity) (Fig. 7b), which was accompanied by an increase in phosphorylation of ACC at Ser^79^ (Fig. 7c). In MI hearts, mAb A treatment resulted in a decrease in AMPK phosphorylation (Fig. 7b), and an attenuation in ACC phosphorylation (Fig. 7c), consistent with the decrease in AMPK activity. In addition, an increase in MCD expression, which degrades malonyl CoA) was seen in the MI hearts, which was prevented by mAb A treatment (Fig. 7d).

### mAb A treatment does not modify post-translational acetylation control of cardiac energy metabolism or ketone oxidation enzyme expression

**Fig. 8:**
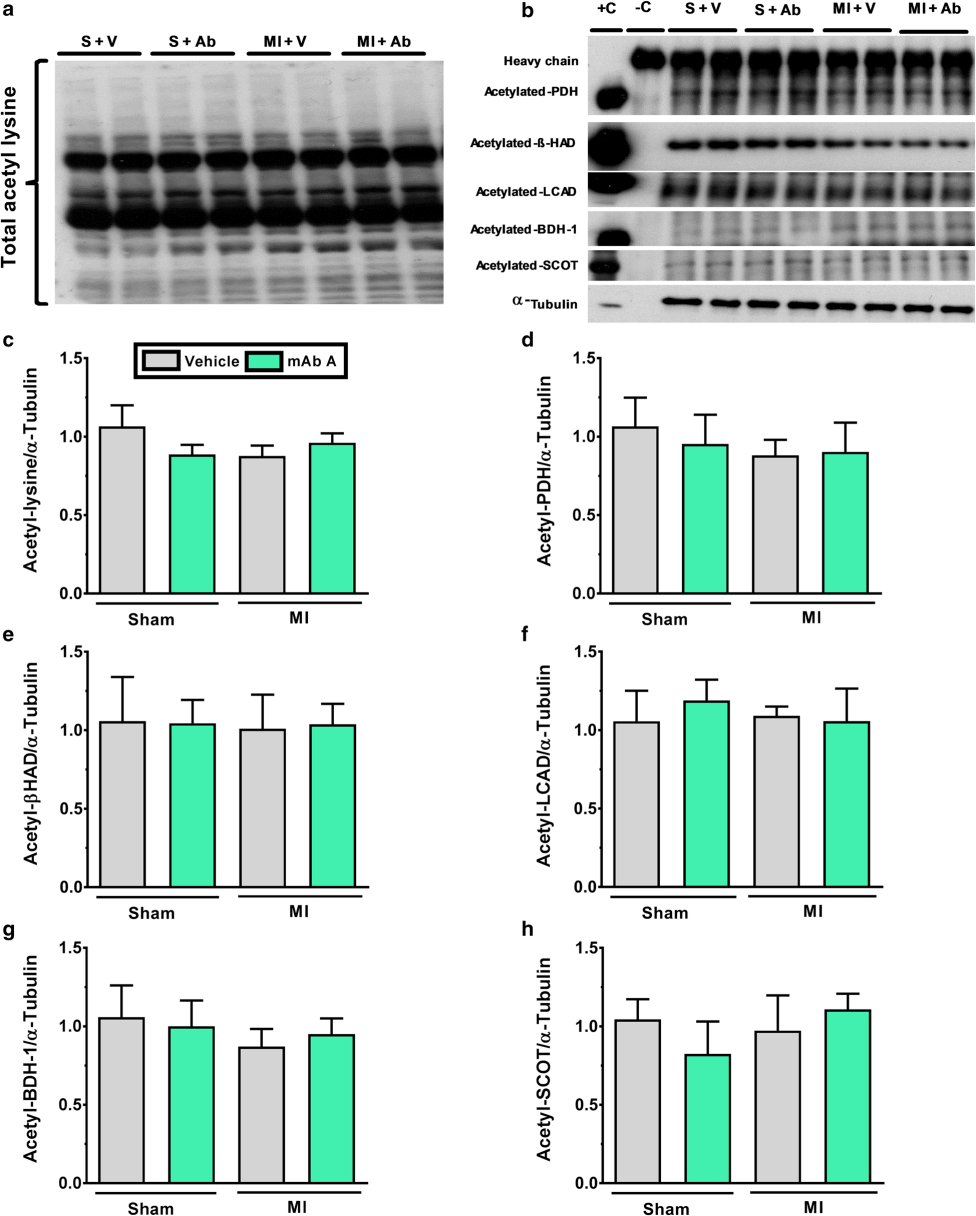
mAb A treatment does not affect the acetylation of the main energy metabolism regulatory kinases and enzymes post-MI. Overall and specific proteins acetylation levels were measured using immunoprecipitation samples. **a** Representative blots for total protein acetylation (acetyl-lysine) for molecular weight range of 250–25 kDa. **b** Representative blots for acetylated-pyruvate dehydrogenase (PDH), acetylated- β-hydroxyacyl CoA dehydrogenase (β-HAD), acetylated-long chain acyl CoA dehydrogenase (LCAD), acetylated-hydroxybutyrate dehydrogenase-1 (BDH-1), acetylated-succinyl-CoA-3-oxaloacid CoA transferase (SCOT) with correspondent α-tubulin as a loading control. Densitometric analysis of **c** total acetyl-lysine, **d** acetylated-PDH/α-tubulin, **e** acetylated-β-HAD/α-tubulin, **f** acetylated-LCAD/α-tubulin, **g** acetylated BDH-1/α-tubulin and **h** acetylated-SCOT/α-tubulin were carried out when protein bands were normalised to α-tubulin bands for intra-experiment variation. Data are reported as mean ± SEM n = 6/group. −*C* negative control, +*C* positive control, *S* sham, *V* vehicle, *Ab* mAb A, *MI* myocardial infarction

Acetylation is a post-translational modification that can modify the activity glucose and fatty acid metabolism regulatory enzymes [47, 48]. Overall protein acetylation in the MI heart was not changed compared to sham group (Fig. 8c). Similarly, the acetylation of PDH, β-HAD and LCAD also were not modified following MI compared to sham hearts (Fig. 8d–f).

**Fig. 9:**
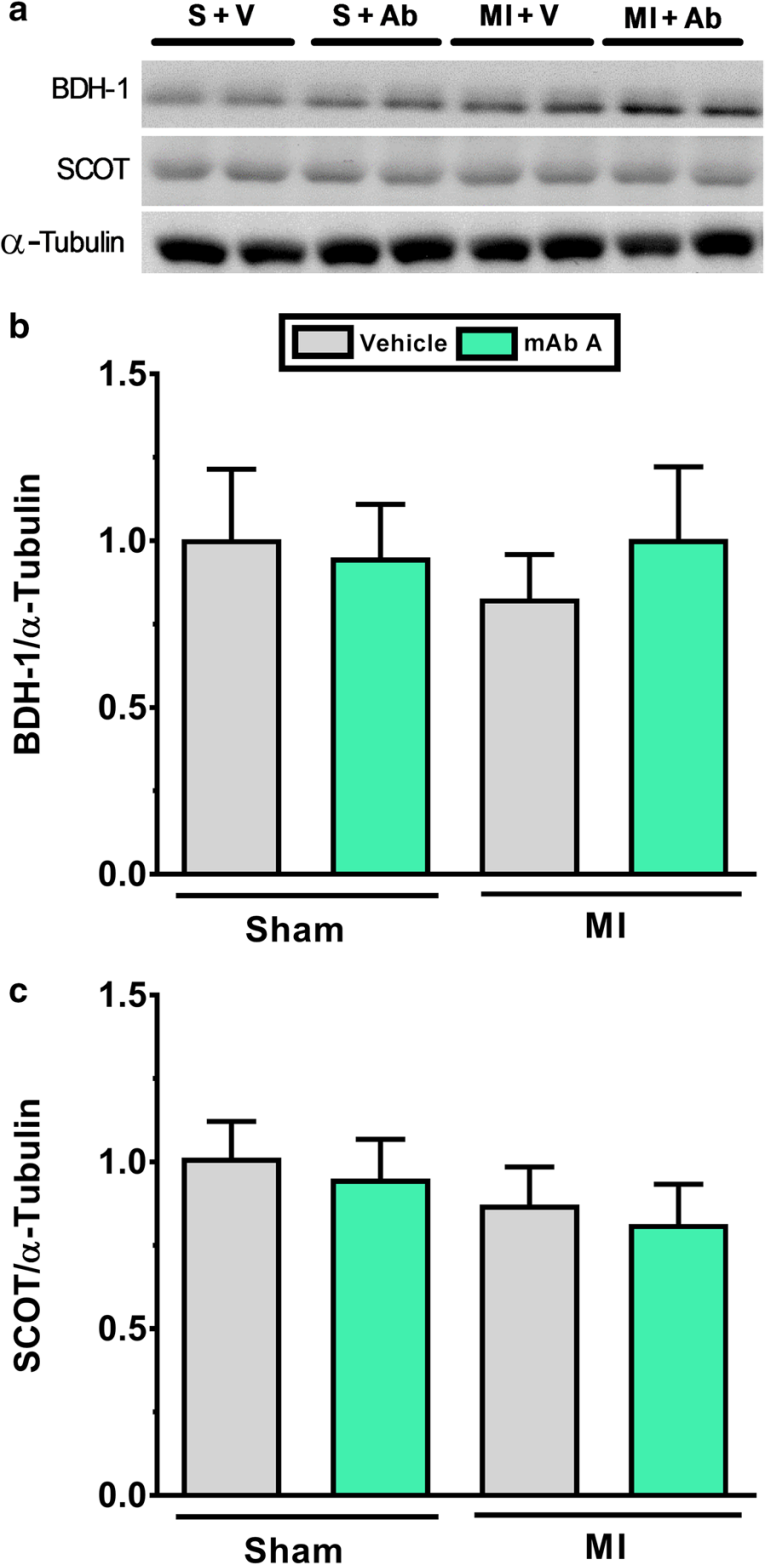
mAb A treatment does not change the expression of ketone oxidation enzymes post-MI **a** representative blots of β-hydroxybutyrate dehydrogenase-1 (BDH-1) and succinyl-CoA-3-oxaloacid CoA transferase (SCOT). Densiometric analysis for **b** BDH-1/α-tubulin, **c** SCOT/α-tubulin. Protein bands were normalised for either to their correspondent total protein band or to α-tubulin bands for intra-experiment variation. *p < 0.05. n = 6/group. *S* sham, *V* vehicle, *Ab* mAb A, *MI* myocardial infarction

We also assessed the expression and the acetylation of ketone oxidation regulatory enzymes, namely β-hydroxybutyrate dehydrogenase-1 (BDH-1) and succinyl-CoA-3-oxaloacid CoA transferase (SCOT). Likewise, we did not detect any change in either the expression (Fig. 9b, c) or the acetylation level (Fig. 8g, h) of these enzymes. Taken together, this indicates that the acetylation level of metabolic enzymes is highly unlikely to play a role in mAb A-mediated cardioprotection and the observed metabolic modifications.

## Discussion

**Fig. 10:**
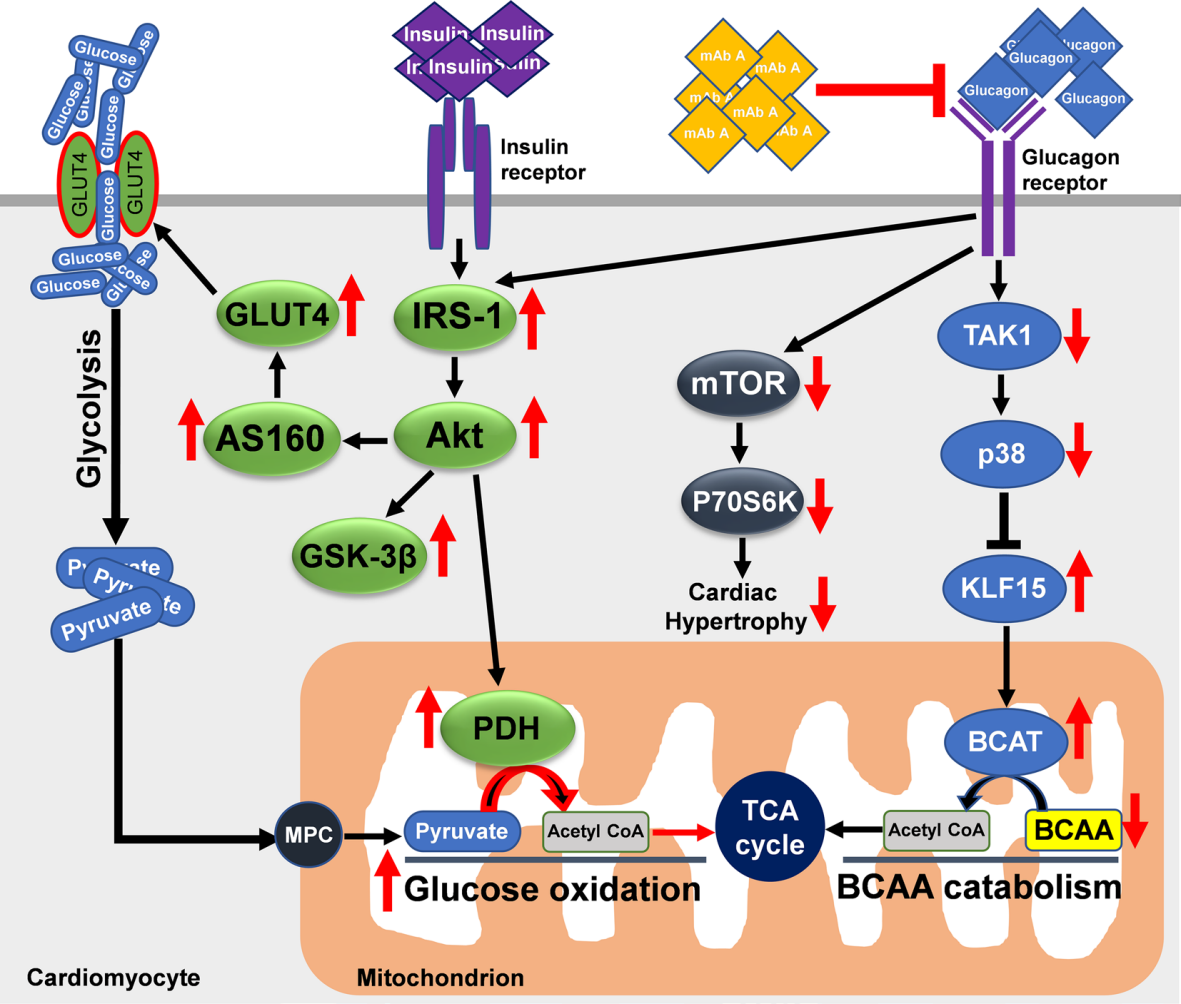
Schematic illustration of the proposed cardioprotective mechanism of mAb A treatment in hearts following a myocardial infarction. Glucagon receptor antagonism increases the insulin signalling pathway and the BCAA catabolic pathway, while inhibiting the mTOR pathway. *IRS*-*1* insulin receptor substrate-1, *Akt* protein kinase B, *GSK*-*3β* glycogen synthase kinase-3 beta, *mTOR* mammalian target of rapamycin, *P70S6K* ribosomal protein S6 kinase, *AS160* Akt substrate 160, *GLUT4* glucose transporter 4, *PDH* pyruvate dehydrogenase, *TAK1* transforming growth factor beta-activated kinase 1, *p38 MAPK* p38 mitogen-activated protein kinase, *KLF15* Krüppel-like factor 15, *BCATm* mitochondrial branched chain aminotransferase, *BCAA* branched chain amino acids, *TCA* tricarboxylic acid, *MPC* mitochondrial pyruvate carrier. Red arrows indicate the cellular changes following mAb A treatment in post-MI heart

This study provides the novel findings that antagonism of the glucagon’s actions, using a pharmacological inhibitor of the glucagon receptor, enhances cardiac function in the post-MI heart and limits adverse cardiac remodelling. Chronic treatment with mAb A improves cardiac insulin sensitivity and enhances insulin-stimulated glucose oxidation in the MI hearts, resulting in an increase in energy production. We also demonstrate that cardioprotection exhibited by glucagon antagonism post-MI is associated with improved BCAA catabolism through a mechanism which involves inhibition of TAK1/p38 MARK signalling and activation of the KLF15/BCATm pathway (Fig. 10). Enhancing BCAA catabolism is also associated with abrogation of mTOR signalling, which not only enhances cardiac insulin signalling but also limits adverse cardiac hypertrophic remodelling post-MI.

### Glucagon antagonism improves cardiac function and insulin sensitivity in the failing heart

Alterations in cardiac energy metabolism in the post-MI heart contribute to the severity of HF development [2, 7]. As a result, optimizing cardiac energy metabolism is a potential therapeutic approach to decreasing the severity of HF and improving clinical outcomes [49]. One approach to optimizing energy metabolism may be to improve cardiac insulin signalling, since the failing heart becomes insulin resistant [2, 7, 50]. Because glucagon opposes insulin signalling, inhibiting glucagon signalling can be one such approach to cardioprotection. In support of this concept, triggering glucagon signalling deteriorates glycemia in diabetes and obesity, both of which are associated with insulin insensitivity [51]. In the heart we demonstrate that blocking glucagon signalling can significantly enhance cardiac insulin sensitivity and enhance insulin-stimulated glucose oxidation which improves cardiac energy production in the MI hearts. Of importance, is that the improvement in cardiac energy production was also associated with improved cardiac function and contractility in vivo and ex vivo. This enhanced glucose oxidation was accompanied by an activation of PDH (the rate-limiting enzymes for glucose oxidation) and the IRS-1/Akt/GSK-3β signalling pathway, a major component of the reperfusion injury salvage kinase (RISK) pathway, which is a cardioprotective signalling pathway post-MI [52]. Our data is supported by previous studies showing that glucagon antagonism mitigates diabetic cardiomyopathy in Lepr^db/db^ diabetic mice, coinciding with improved whole body insulin signalling [53]. Antagonising glucagon signalling in patients with type 1 [54] and type 2 [55] diabetes also improves glycaemic control and reduces daily insulin requirements, suggesting enhanced insulin sensitivity and signalling. Of interest, is that emerging therapeutic approaches of metabolic disease have recently shown to improve insulin sensitivity and cardiac function in type 2 diabetic patient and experimental models. For example, Ramirez et al. [56] demonstrated that glucagon-like peptide-1 (GLP-1) enhancer, sitagliptin, enhanced diastolic function and cardiac efficiency in type 2 diabetic rats by shifting fatty acid to glucose utilization in cardiomyocytes and reducing lipolysis. A concomitant therapy of GLP-1 receptor agonist (GLP-1 RA) along with conventional hypoglycemia treatment is also shown to improve cardiac function along with reduction in systematic inflammation and circulating BNP levels in cardiac resynchronization therapy with a defibrillator in failing heart patients [57]. Furthermore, another multi-target approach using a hybrid compound (GLP-1/xenin) has also been shown to improve glycemic control and reduce circulating lipid in a mouse model of obesity-induced type 2 diabetes [58]. Therefore, a combination therapy could be a potential therapeutic approach to attenuate insulin resistance and enhance cardiac function in type 2 diabetic patients. Taken together, enhanced cardiac insulin sensitivity by antagonising GCGR in our study might be, in part, secondary to the enhancement of the whole-body insulin sensitivity which could have significant implications in the view of the ongoing debate about localization and abundancy of GCGR in the heart [59].

### mAb A treatment-mediated cardioprotection post-MI is associated with enhanced BCAA catabolism

It has been shown that glucagon administration increases mortality in mice in vivo and impairs functional recovery in vitro following a LAD coronary artery ligation via p38 MAPK-dependent manner [32]. In this study, the phosphorylation of p38 MAPK was increased post-MI along with its upstream transcriptional regulator, TAK1. This activation of p38 MAPK was associated with a marked decrease in KLF15 as well as BCATm expression in the myocardium, suggesting an impaired BCAA catabolism. This notion is further supported by a marked accumulation of cardiac BCAA post-MI with no change in the levels of circulating BCAA, which is consistent with an impaired BCAA catabolism in the MI heart. However, mAb A treatment significantly abrogated TAK1/p38 MAPK signalling and preserved BCATm activity which was accompanied by a decrease in cardiac BCAA accumulation post-MI. We therefore propose that glucose receptor antagonism increases cardiac insulin stimulated glucose oxidation in part by stimulating BCAA catabolism (Fig. 10).

### Cardioprotection established by mAb A treatment in the post-MI hearts was accompanied by inhibition of AMPK signalling pathway

We also investigated how mAb A treatment prevents MI-induced pathological hypertrophy and improves cardiac contractility in vivo and ex vivo post-MI. This prevention of adverse cardiac remodelling post-MI was associated with the inhibition of the mTOR/P70S6K signalling pathway in the myocardium. Consistent with this observation, previous work by Ali et al. [32] demonstrated that cardiac-specific deletion of glucagon receptor (*Gcgr*^*CM*−*/*−^) caused a reduction in infarct size following a permanent ligation of the LAD compared to wild type mice, emphasizing the possible negative role of glucagon in the myocardium post-MI. Of interest, is that the infarct-limiting effect by *Gcgr*^*CM*−*/*−^ was associated with inhibition of fatty acid oxidation [32]. High fatty acid oxidation rates can also adversely affect heart function post-MI [44–46, 49]. This occurs, in part due to an inhibition of glucose oxidation [3, 4, 7, 60]. Interestingly, mAb A treatment markedly decreased cardiac fatty acid oxidation in the MI mice (Fig. 4b). As a result, mAb A resulted in a significant shift in cardiac energy metabolism from fatty acid oxidation to glucose oxidation (Fig. 4d). We propose that this shift contributed to the beneficial effect of mAb A in the MI hearts. This was further supported by inhibition of AMPK along with activation of ACC and reduction in MCD expression (Fig. 8), main regulators of malonyl CoA-mediated inhibitory effect on fatty acid oxidation [61], which would be expected to increase malonyl CoA levels, thereby decreasing fatty acid oxidation rates.

### The effect of mAb A treatment on cardiac ketone signalling and oxidation

Ketones have attracted considerable recent attention recently as a potential “thrifty fuel” in the failing heart [13, 15]. Our study is the first to directly measure ketone oxidation rates in the heart post-MI. Curiously, MI caused a significant reduction in ketone oxidation rates and the contribution of ketone oxidation to ATP production. In addition, mAb A treatment actually further decreased ketone oxidation in hearts from MI mice. These alterations in ketone oxidation were not accompanied by changes in the expression of either BDH-1 or SCOT post-MI. This may suggest that changes in ketone oxidation are secondary to the enhanced glucose oxidation rates and its contribution to acetyl CoA production for the TCA cycle. It may also indicate, at least in the post-MI heart, that the elevation in the circulating level of ketones (i.e. ketosis) is an attempt to compensate for the cardiac energy crisis which is not necessarily accompanied with an improvement in cardiac function. In support of this, we recently reported that empagliflozin, a sodium glucose cotransporter-2 (SGLT2) inhibitor that induces ketosis, improves cardiac function in *db/db* diabetes mice [62]. This cardioprotection was in fact accompanied by an improved cardiac energy production from glucose and fatty acid oxidation, but not ketone oxidation [62]. In support of this, another recent study demonstrated that in diabetic hypertensive rats, empagliflozin treatment preserved glucose utilization and reduces cardiac afterload without a significant effect on myocardial ketone utilization despite increased circulating levels of ketones [63]. Taken together, it seems plausible to suggest that ketone is an extra source of energy for the heart and that the increased in ketone levels can enhance energy production and function, but that neither the presence of an MI or treatment with mAb A directly increases cardiac ketone oxidation rates in the heart.

### Limitations

Our current studies have a number of caveats which need to be acknowledged. First, we employed a young, healthy, nondiabetic and no obese mice to develop an in vivo model of myocardial infarction. However, whether the same extent of cardioprotection by mAb A treatment could be seen in aged, diabetic or obese mice with myocardial infarction has yet to be investigated. Second, our main focus in this study was to characterise the effect of mAb A treatment on cardiac energy metabolism and insulin sensitivity post-MI in parallel with cardiac glucose oxidation and BCAA metabolism. Nevertheless, we have not evaluated the role of indirect effect(s) from other organs/tissues on cardiac energy metabolism and insulin signalling. Thus, enhanced insulin sensitivity of the whole body by mAb A treatment might have contributed to the demonstrated improvement in cardiac insulin sensitivity and function in the MI hearts. In addition, improved BCAA catabolism may play an important role in mediating the effect of mAb A on cardiac insulin signalling in the MI hearts. However, whether the decrease in the level of cardiac BCAA can be directly attributed to increased BCAA oxidation in the MI hearts post mAb A treatment has yet to be determined, and is an important scope for future investigations. Furthermore, it also remains unclear whether the reduction in ketone oxidation by mAb A treatment in MI hearts is due to the reduction in ketone uptake by the heart as a competitive mechanism in response to the up-regulation of GLUT4.

## Conclusions

Optimizing cardiac energy production in the failing heart via improving cardiac insulin signalling is cardioprotective against cardiac dysfunction and hypertrophy. Pharmacological antagonism of glucagon signalling is an effective cardioprotective maneuverer to optimize cardiac energy production via enhancing cardiac insulin signalling and insulin-stimulated glucose oxidation post-MI.

## Abbreviations

ACC: acetyl CoA carboxylase
Akt: protein kinase A
AMPK: 5′ AMP-activated protein kinase
BCAA: branched chain amino acids
BCATm: mitochondrial branched chain aminotransferase
BDH-1: beta-hydroxybutyrate dehydrogenase-1
β-HAD: beta-hydroxyacyl CoA dehydrogenase
β-OHB: beta-hydroxybutyrate
CPT-1β: carnitine palmitoyltransferase-1 beta
GCGR: G-protein coupled glucagon receptor
GLUT4: glucose transporter type 4
HF: heart failure
IRS-1: insulin receptor substrate 1
KLF15: Krüppel-like factor 15
LCAD: long chain acyl CoA dehydrogenase
MI: myocardial infarction
p38 MAPK: p38 mitogen-activated protein kinases
PDH: pyruvate dehydrogenase
PPAR-α: peroxisome proliferator-activated receptor-alpha
SCOT: succinyl-CoA-3-oxaloacid CoA transferase
TAK1: transforming growth factor beta-activated kinase 1
TCA: tricarboxylic acid
